# Quintuple labeling in the electron microscope with genetically encoded enhanced horseradish peroxidase

**DOI:** 10.1371/journal.pone.0200693

**Published:** 2018-07-16

**Authors:** Didiana Cruz-Lopez, Dianne Ramos, Gloria Castilloveitia, Thomas Schikorski

**Affiliations:** Department of Neuroscience, Universidad Central del Caribe, Bayamon, Puerto Rico, United States of America; National Eye Centre, UNITED STATES

## Abstract

Genetic encoded multilabeling is essential for modern cell biology. In fluorescence microscopy this need has been satisfied by the development of numerous color-variants of the green fluorescent protein. In electron microscopy, however, true genetic encoded multilabeling is currently not possible. Here, we introduce combinatorial cell organelle type-specific labeling as a strategy for multilabeling. First, we created a reliable and high sensitive label by evolving the catalytic activity of horseradish peroxidase (HRP). We then built fusion proteins that targeted our new enhanced HRP (eHRP) to three cell organelles whose labeling pattern did not overlap with each other. The labeling of the endoplasmic reticulum, synaptic vesicles and the plasma membrane consequently allowed for triple labeling in the EM. The combinatorial expression of the three organelle-specific constructs increased the number of clearly distinguishable labels to seven. This strategy of multilabeling for EM closes a significant gap in our tool set and has a broad application range in cell biology.

## Introduction

The introduction of genetically encoded fluorescent proteins has revolutionized cell biology. Since then, there has been an ever increasing need for multicolor labeling of individual samples. In fluorescence microscopy, this need motivated the continuous development of new, brighter, and differently colored fluorescence proteins. This development eventually culminated in the Brainbow construct [1] that allows for multilabeling with up to 100 different genetically encoded colors. Electron microscopy, however, lagged behind these developments. Not because that there is no need for such technologies but because it has been proven difficult to find a suitable genetically encoded label.

Several attempts to introduce a genetically encoded marker for EM have been made in the past. LacZ/ß-galactosidase was the first, but the signal in the EM is of low contrast and diffuses across cell membranes which convolute the identification of labeled structures. Genetically encoded fluorescent markers that are photoconvertable also have been used for EM. To this group belongs ReAsH that binds to a recombinant tetracysteine motif [2,3] and the small fluorescent proteins called minisogs [4]. A second strategy for the use of fluorescent labels has been the alignment of high magnification fluorescent images with EM graphs for the identification of labeled structure in correlated light and electron microscopy [5,6]. But such a strategy does not truly qualify as a genetically encoded label for EM because an actual label in the EM does not exist. The major drawbacks of these fluorescence based techniques are that they are only applicable to small areas of interest and are not suitable for multilabeling. The most recent approach has been the genetically engineered ascorbate peroxidase variant APEX [7] that has overcome some of the limitations of fluorescent based techniques. However, APEX is of low contrast in the LM and EM although contrast improved with APEX2 [8]. Undoubtedly, these genetically encoded labels are a significant step forward, but more advances are needed before genetic approaches in the EM can catch up with existing fluorescent techniques. In particular, improvements in detection sensitivity are needed. More so, because EM is inherently monochrome, new labeling strategies must enable true multilabeling with five or more genetically encoded labels.

In this study, we explored whether cell-organelle specific labeling might be a useful and feasible strategy for multilabeling in the EM. To implement this strategy, we first evolved horseradish peroxidase’s (HRP) catalytic activity and generated a genetically encoded label for EM whose sensitivity exceeds APEX2 in the EM and eGFP in the LM. We, then, targeted enhanced HRP (eHRP) to the endoplasmic reticulum (ER), synaptic vesicles (SV), and the plasma membrane (PM). This strategy resulted in three clearly distinguishable labeling patterns in the EM. We accomplished our goal of true multilabeling through combinatorial expression of all three labels. This approach resulted in seven unique labels within a single specimen.

## Materials and methods

All experiments were approved by the Animal Care and Use Committee of the Universidad Central del Caribe and conformed to the regulations of the National Institute of Health guidelines for the use of laboratory animals.

### HRP mutants

The following variants of the HRPc isoenzyme were a kind gift from Dr. Frances Arnold (California Institute of Technology, Pasadena, CA, USA): WT, H1-8H10, H1-13A10, 10G5, H1-6E1, and 13A10_N175S [9]. The new variants were generated using StEP recombination with the following templates: H1-8H10, H1-6E1, H1-10G5, and WT. Further mutations were introduced via site-directed mutagenesis. All primers for site-directed mutagenesis were designed with the help of Qiagen’s online tool (Qiagen Inc., Venlo, The Netherlands).

For screening, the variants were targeted to the ER and cloned into mammalian expression vectors (pDC515, Microbix Biosystems Inc., Ontario, Canada and pIRES2eGFP, Clontech Laboratories Inc., Mountain View, CA, USA). The signal sequence of the glutamate receptor-4 (GluR4) [10] preceded HRP and was built by assembly polymerase chain reaction (PCR) with the following primers:

ACGAATGGATCCGCCACCATGAGGATTATTTGCAGGCAGATTG, CGAGTCCCCAAAATCCAGAAAACAACAAGACAATCTGCCTGCAAATAATCCTC, TTTCTGGATTTTGGGGACTCGCCATGGGACAGTTAACCCCTACATTCTACGAC, and GGACACGTTGGGACAGCTATTGTCGTAGAATGTAGGGGTTAACT together with the two flanking primers ACGAATGGATCCGCCACC and GGACACGTTGGGACAGC. The The PCR assembly product contained the first 42 base pairs of HRP and was cloned in front of HRP by using HRP’s AFLIII site at position 38. The ER retention signal, KDEL, was added downstream of HRP. This GluR4HRPKDEL chimera was flanked with EcoR1 and BamHI restriction sites for routine subcloning.

### Cell culture and HRP mutant transfection

The neuroblastoma cell line (NB41a, ATCC, Manassas, VA, USA) was propagated in Neurobasal-A medium supplemented with B27-supplement (Life Technologies, Carlsbad, CA, USA). Hippocampal cell cultures were prepared from newborn rat pups as described previously [11,12].

NB41a cells were transfected with Lipofectamine 2000 (Life Technologies). Neurons were transfected with standard calcium phosphate techniques on day 1 after plating. Lentiviral vectors were prepared in human embryonic kidney cells (HEK, ATCC, Manassas, VA, USA) by co-transfection with pMDLg/pPRE, pRSV-Rev, pCMV-VSV-G, and pFUGW-H1 vectors (all from addgene.org, HRP was cloned into pFUGW-H1). Three days after transfection, the medium containing viral vector, was collected and added to neuronal cultures. The titer of viral particles was not determined and fluctuates among different preparations. Increasing amounts of viral particles were induced by doubling and tripling the amount of viral media from the same preparation added to neuronal cultures.

### Fixation and HRP histochemistry

Samples were fixed in 4% paraformaldehyde, 0.125% glutaraldehyde, or 2.5% glutaraldehyde in HEPES-buffered saline for 5–10 min. Specimens were washed in Tris-buffered saline (TBS, pH 7.2–7.5) and developed in 0.05% 3,3-diaminobenzidine (DAB; Kem-En-Tec Diagnostics, Taastrup, Denmark) in TBS and 0.0003–0.006% hydrogen peroxide. Development times varied from 10–240 minutes (usually 30–60 min). All samples were postfixed and stored until processing for EM in 4% paraformaldehyde and 5% glutaraldehyde in cacodylate buffer (pH 7.4). For details, see [12]).

Images were acquired with a Nikon FN1 epifluorescence microscope and Nikon NIS software package (Nikon, Tokyo, Japan).

### Electron microscopy

Selected specimens of NB41 cells and cultured hippocampal neurons were processed for EM as previously described (Schikorski, 2010). EM graphs were acquired with a digital camera (Hamamatsu 4MP) and AMT software by using a JEOL 100CX transmission electron microscope (JEOL Ltd., Tokyo, Japan). All EM graphs were shading corrected in real time.

### Image analysis

Images were not enhanced with the exception of balancing brightness and contrast by using the dodging and burning tools in Adobe Photoshop (Adobe Inc., San Jose, CA, USA).

For densitometry measurements of cell bodies in LM, acquired images were shading corrected and the average pixel values of cell bodies were measured by outlining the cell body with ImageJ’s “arbritary area” tool (National Institutes of Health, Baltimore, MD, USA). Pixel values were converted to transmittance values by dividing average pixel values of individual cells by the incident light (a pixel value of 255 was used for incident light). Transmittance was converted to density by using the equation: density = 2*log (1/transmittance).

## Results and discussion

### Directed evolution of HRP

Our rationale for creating an advanced HRP was to elevate HRP’s catalytic activity such that the elevated activity will overcome existing limitations of HRP as a genetically encoded marker in the LM and EM.

The catalytic activity of the HRPc isoenzyme has been previously evolved in yeast [9,13]. For our purpose, these mutants were a logical starting point, and we selected several mutants that had at least a 100-fold higher catalytic activity than wild-type (WT) HRP when expressed in yeast. We cloned these mutants into mammalian expression vectors for the expression in mammalian cells. Each HRP mutant was flanked upstream with the signal peptide of the glutamate receptor 4 (GluR4) and downstream with the ER retention signal, KDEL. When expressed in mammalian cells, GluR4_HRP_KDEL protein was retained inside the ER, although small amounts could also be found at the *cis*-stacks of the Golgi apparatus [14].

**Fig 1:**
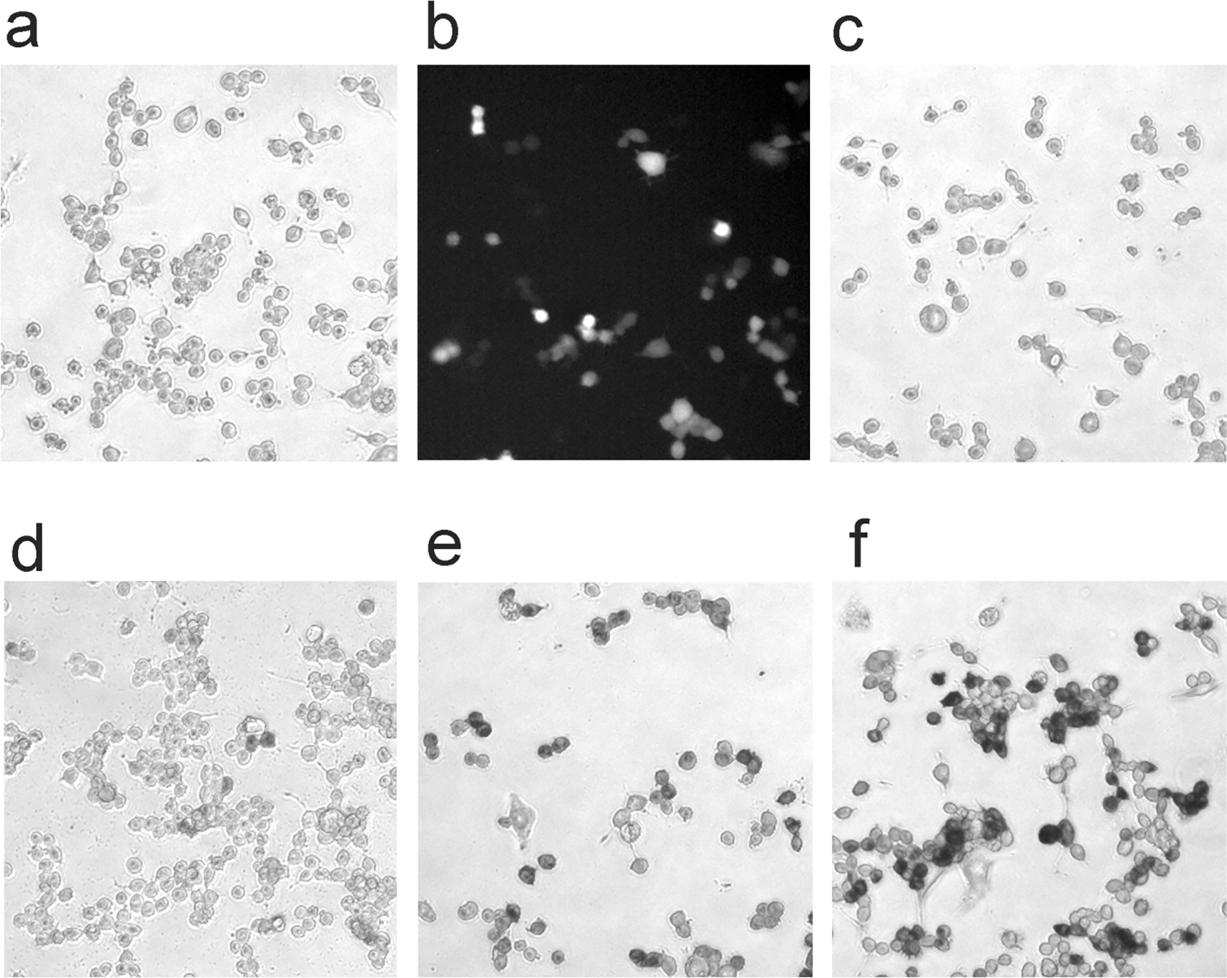
Expression of horseradish peroxidase variants in neuroblastoma cells. (a) WT HRP transfection did not result in detectable HRP reaction product. (b) eGFP was reliably detected. Most HRP variants with enhanced catalytic activity were HRP-positive in bright field images: (c) 13A7_N175S), (d) H1-8H10, (e) H1-6E1, and (f) H2-10G5_M83I.

For routine screening of many mutants, we employed human embryonic kidney cells and because of our interest in neuronal applications, we also used the neuronal cancer cell line NB41a. NB41a cells can routinely be propagated and were easily transfected in high numbers with eGFP (Fig 1B). WT HRP expression in NB41a cells, on the other hand, was as problematic as in neurons (Fig 1A). Very few cells if any exhibited the typical brown label resulting from WT HRP cytochemistry. Therefore, NB41a cells were a good model for screening HRP mutants for their suitability for neuronal expression. We also noticed that all mutants that produced a strong signal in NB41a cells always produced a superior signal in other mammalian cells. Therefore, we focused in many cases on NB41a cells for the directed evolution of HRP.

When transfecting NB41a cells with HRP mutants, HRP-positive cells became detectable after standard HRP cytochemistry (Fig 1D–1F). Surprisingly, one mutant (13A7_N175S; Morawski et al., 2001) that had very high activity, high thermal stability, and was most suitable for work in yeast did not produce better labeling in NB41a cells than WT HRP (Fig 1C; for nomenclature see [9] and Table 1). The high-activity mutants H1-6E1 and H1-8H10 did show some improvement over WT HRP (Fig 1D and 1E), but HRP-positive NB41a cells were most easily detected with the H2-10G5_M83I variant (Fig 1F). Apparently some mutations result in elevated activity in one cell type (e.g., yeast), while the same mutations do not enhance activity in other cell types.

**Table 1:** List of HRP variants and their corresponding mutations.

Mutation	M83I	R93L	T102A	L131P	N175S	N212D	L223Q	K232E	V303E
**13A7_N175S**	-	X	X	X	X	X	-	-	X
**8H10**	-	X	X	X	-	X	X	-	X
**13A10**	-	X	X	X	-	-	X	-	X
**6E1**	-	X	X	X	-	-	X	X	X
**10G5**	X	X	X	X	X	X	X	-	X
**13A5LE**	X	-	-	X	-	-	X	X	X
**eHRP**	X	-	-	X	-	X	X	X	X

If this interpretation was correct, a new combination of already known mutations may result in a superior HRP variant for mammalian and neuronal expression. For the creation of new variants, we used StEP recombination with WT, H1-6E1, H1-10G5, and 13A10. When screening the new mutants in NB41a cells, one mutant produced noticeably higher-density labeling than others. Sequencing revealed that the mutant contained the following five mutations: L131P, N135, L223Q, K232E, T257, and V303E. To further enhance this mutant’s activity, we changed the methionine in position 83 to the hydrophobic amino acid leucine (N83L), a point mutation previously shown to increase HRP’s catalytic activity [9]. Because this mutant contained five mutations from the original 13A10 mutant plus the K232E mutation inherited from the 6E1 mutant and the M83L mutation, we called this mutant 13A5LE. The 10G5 mutant, which worked best among the original mutants in NB41a cells, also contained a change of the asparagine at position 212 to an aspartate (N212D), therefore, we introduced this mutation into 13A5LE. For a complete summary of the mutations see Table 1.

### Quantifying the labeling intensity of HRP variants

**Fig 2:**
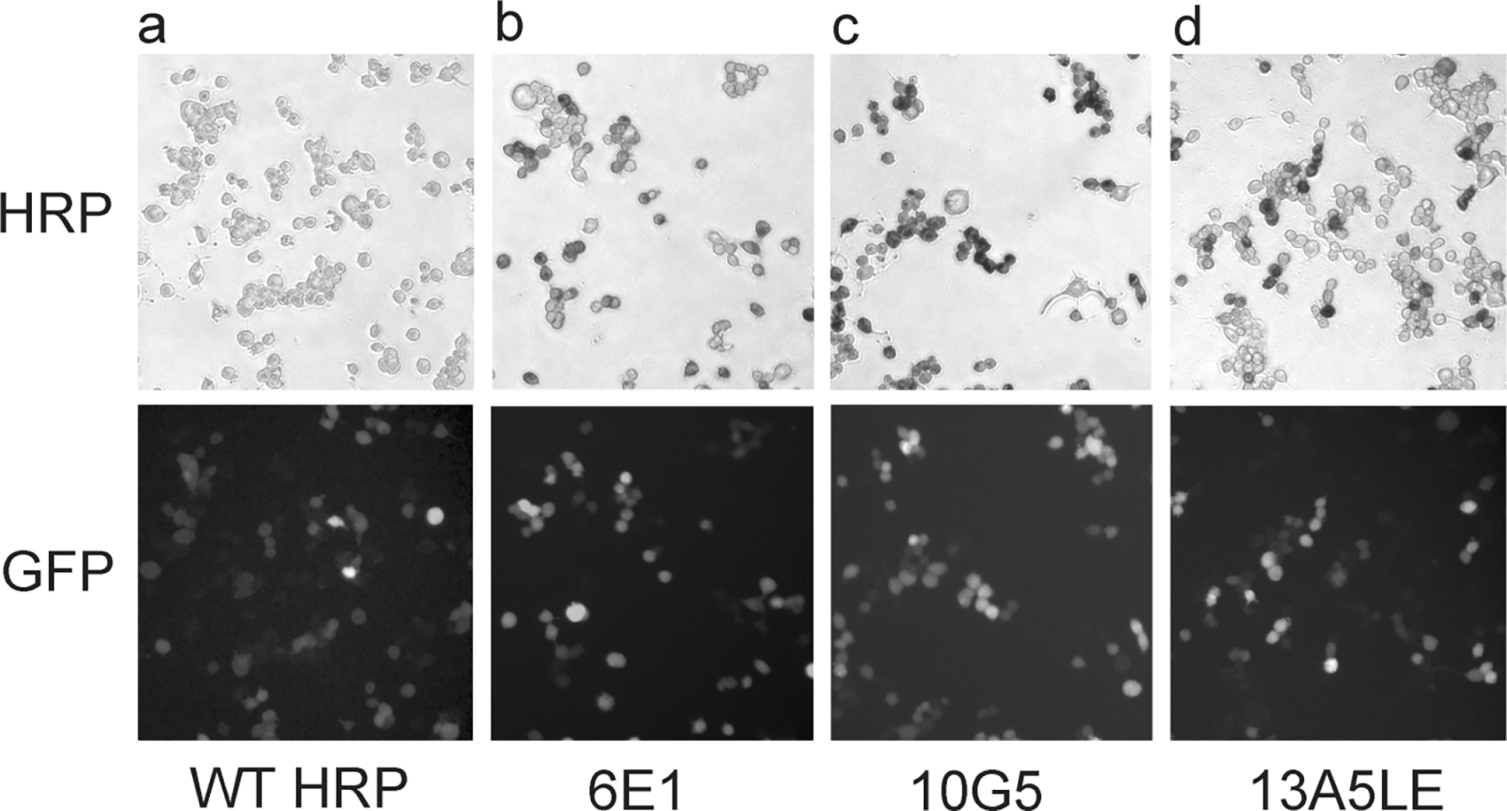
Co-transfection of NB41a cells with eGFP and various HRP mutants. The top row shows the bright-field images after HRP cytochemistry, and the bottom row depicts the corresponding epi-fluorescence image (eGFP).

**Fig 3:**
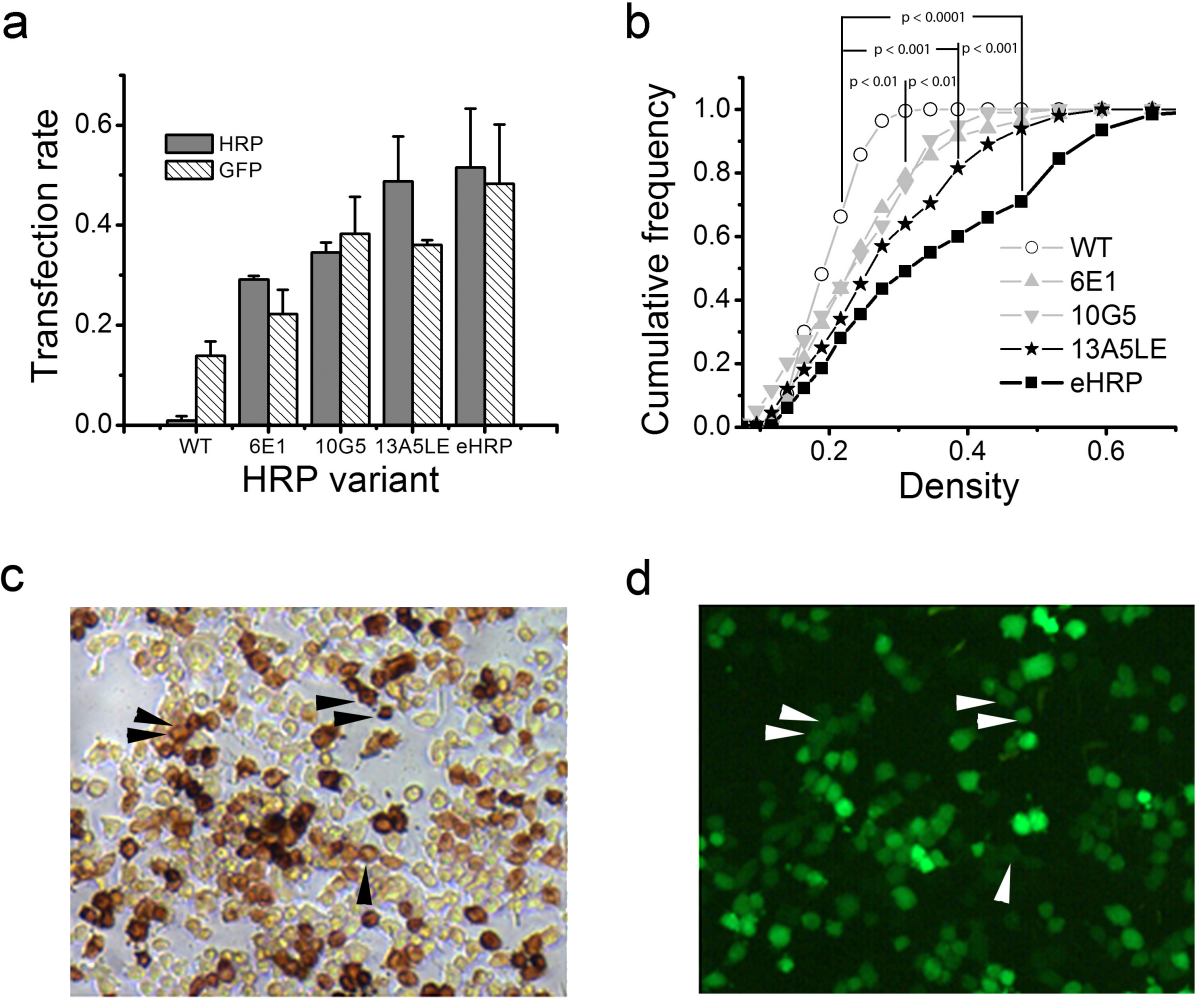
Comparison of HRP variants targeted to the endoplasmic reticulum (ER). (a) Comparison of transfection rates after HRP and eGFP co-transfection in neuroblastoma cells. Only the co-expression of WT HRP and eGFP was significant different (t-test p 0.001). (b) Cumulative histogram of the density of neuroblastoma cell body transfected with various HRP mutants. eHRP produced the highest densities. Both 10G5 and 6E1 were significantly different compared to WT and 13A5LE was significantly different to 10G5 and 6E1. eHRP produced the highest densities. The Kolmogorov-Smirnov test was used to determine significant differences. Comparison of eHRP (c) with eGFP (d). The black arrowheads point to examples where the eHRP density produces a stronger signal than eGFP (white arrowheads in (d)).

To compare the new HRP variants with eGFP, we cloned the various HRP variants into the pIRES2eGFP vector for co-expression of HRP mutants and eGFP. In these experiments, each sample was imaged twice, first under epi-fluorescence and again in bright-field after fixation and HRP cytochemistry (Fig 2). As expected, co-transfection of eGFP and WT HRP resulted in numerous fluorescent cells but not in HRP-positive cells (Fig 2A). When eGFP was co-transfected with high-activity HRP variants, all eGFP-positive cells were also HRP positive (examples are shown in Fig 2B–2D). The fraction of eGFP- to HRP-positive cells was significantly different for WT HRP (p < 0.01) but not different for HRP mutants with high catalytic activity (Fig 3A).

In these experiments, it was evident that not only more cells carried the HRP label but also that the densities of the HRP reaction product varied among HRP mutants. The most likely reason for this is a difference in catalytic activity; therefore a quantification of the density of the HRP reaction product may allow further characterization of the new HRP variants. The cumulative histogram of the densities of neuroblastoma cells transfected with WT-HRP targeted to the ER contained only HRP-negative cells that had densities below 0.28 (Fig 3B). Cells expressing HRP mutants previously developed in yeast exhibited a tail toward higher densities of up to 0.4. Our new HRP variants were superior; the reaction product reached densities beyond 0.6, a significant increase compared to previous mutants. Considering that HRP was targeted to the ER and that the cytosol itself was not labeled, such a strong increase in density is particularly noteworthy. We named the HRP mutant that produced the highest densities enhanced HRP (eHRP).

Finally, we compared eHRP and eGFP labeling in the same samples. As depicted in Fig 3C and d all eGFP positive cells were also eHRP positive. More so, eHRP labeled many cells more intensely than eGFP although eGFP was expressed in the cytosol and eHRP only in the ER. The experiments suggest that eHRP detection is in some experiments more reliable than eGFP fluorescence.

### eHRP expression in hippocampal neurons

**Fig 4:**
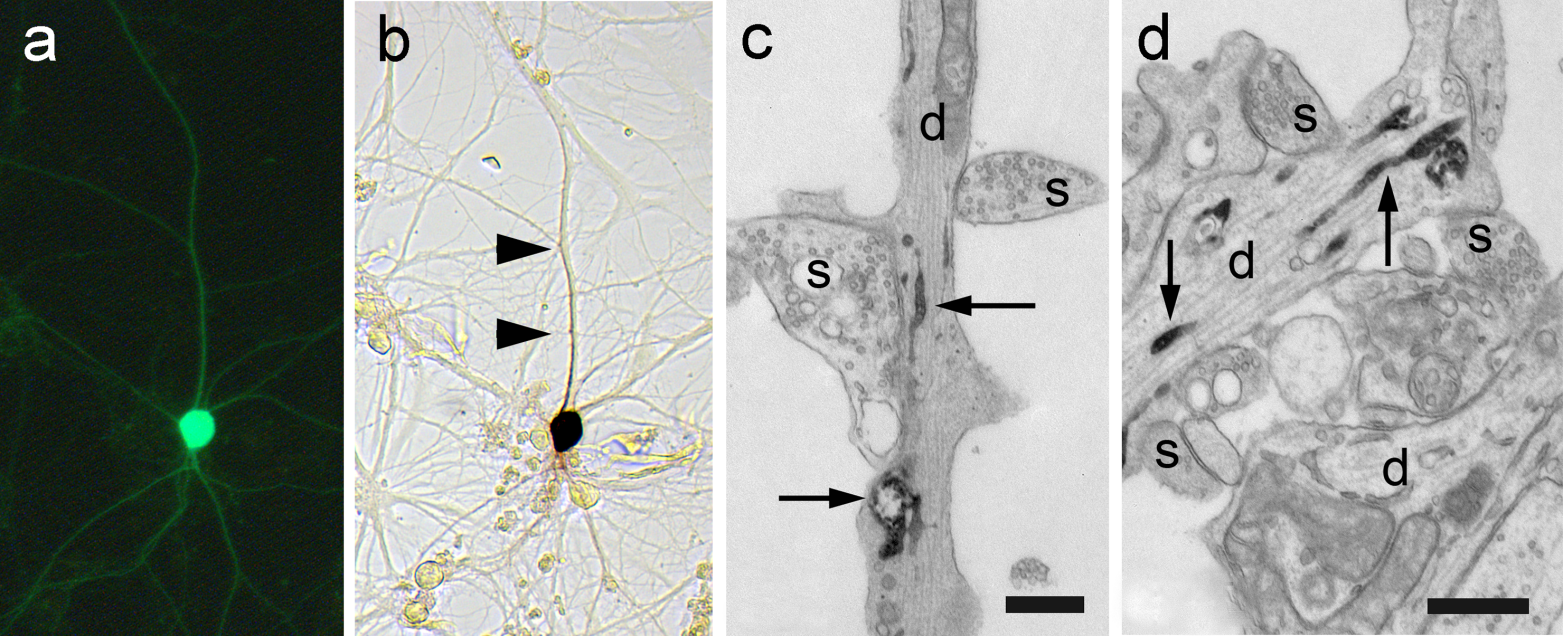
Live imaging correlated with light-electron microscopy. Cultured hippocampal neurons were transfected with eGFP (a) and ER-targeted eHRP (b). The arrowheads point to the HRP-labeled dendrite. (c) Electron micrograph of the same HRP-labeled dendrite as in (b). The ultrastructure is optimally preserved. The arrows point to the labeled ER. (d) Unlabeled (bottom) and labeled (top) dendrites when imaged in the EM. Note the high signal-to-noise ratio of the HRP-positive ER (arrows) compared to the ER in the unlabeled dendrite (d). Both dendrites are innervated by presynaptic boutons (s). Bars in (c) and (d) = 500 nm.

To assess eHRP’s functionality in mammalian neurons, we co-transfected cultured hippocampal neurons with eGFP and eHRP targeted to the ER by again exploiting the IRES sequence (Fig 4). In 23 experiments, 50 neurons were eGFP positive in live imaging sessions and 42 neurons were also found HRP-positive after standard HRP cytochemistry. Of the remaining 8 neurons, 6 were lost during fixation and/or HRP cytochemistry and two showed ambiguous HRP label in the LM.

Next, we tested the suitability of eHRP for correlative live imaging, LM, and EM. We co-transfected cultured hippocampal neurons with GluR4_eHRP_KDEL and eGFP. eGFP fluorescence in neurons was imaged in live sessions (Fig 4A) and after fixation and HRP cytochemistry in bright field microscopy (Fig 4B). After processing for EM, the same labeled neuron and all its processes were easily and reliably identified in the EM (Fig 4C and 4D). The localization pattern of ER-targeted eHRP reaction product was as previously reported for WT HRP [14]. The signal-to-noise ratio, however, was much enhanced as expected from our densitometry measurements, such that HRP-positive neuronal processes were easily and unambiguously identified during EM imaging sessions. These experiments show that, co-expression of eGFP and eHRP is a persuasive strategy for correlative live imaging—light-electron microscopy.

### Epi-fluorescence imaging of eHRP expression

**Fig 5:**
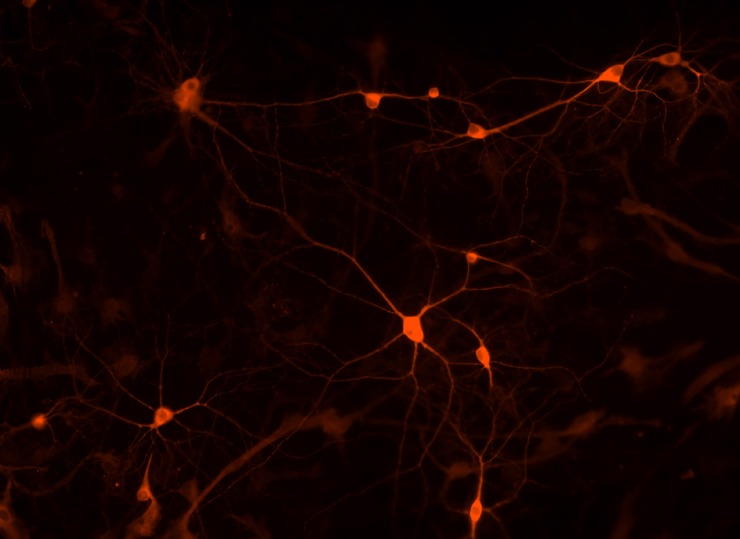
High resolution fluorescent image of eHRP labeled neurons in culture. eHRP was targeted to the endoplasmic reticulum and visualized with Amplex Red.

Although DAB is the classic HRP substrate, AmplexRed that generates a red fluorescent HRP reaction product offers the opportunity for high resolution fluorescent microscopy with eHRP. After lentiviral infection of cultured hippocampal neurons with GluR4eHRPKDEL eHRP positive neurons were visualized with AmplexRed (Fig 5). Numerous neurons were eHRP positive and although eHRP was localized only in the ER the entire neuronal morphology was revealed which once again demonstrated the outstanding sensitivity of eHRP.

### Versatility of eHRP for cell biological applications

To test the versatility of eHRP for various approaches in cell biology, we build additional eHRP fusion proteins. To access the dimensions of complex cells with all their processes and filopodia; it is often more advantageous to label the cell surface rather than the cytosol. This approach is also preferred for EM because the intracellular fine structure will not be compromised by the HRP reaction product. We created an eHRP fusion protein that used the transmembrane domain of the fibronectin receptor and placed eHRP at the luminal side of secretory vesicles such that after insertion into the plasma membrane, eHRP would reside on the cell surface.

**Fig 6:**
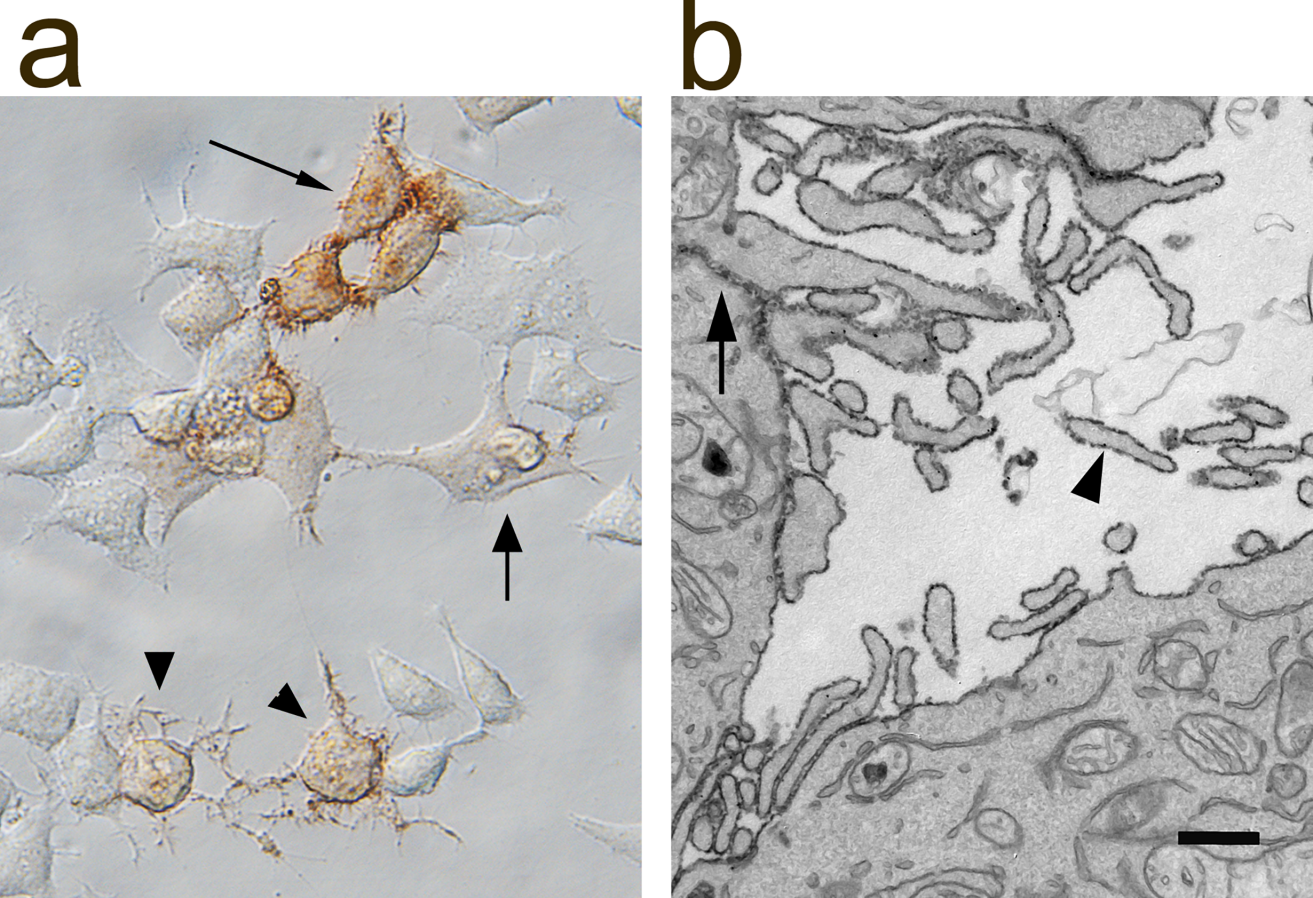
Targeting eHRP to the plasma membrane. (a) Neuroblastoma cells transfected with eHRP targeted to the plasma membrane in the light microscope. In eHRP positive cells many more fine processes can be resolved. (b) Electron micrograph of two neighboring eHRP positive cells. eHRP reaction product is found evenly distributed on the extracellular side of the plasma membrane. Even on the finest filopodia (arrowhead) are labeled. The arrow points to the thin labeled extracellular space between a process and a cell body. Bar = 300 nm.

After transfection into NB41a cells eHRP labeling of the cell surface was already obvious in the LM (Fig 6A). After processing for EM, it was evident that eHRP was correctly targeted to the plasma membrane (Fig 6B) and serial sectioning confirmed that the entire surface of transfected NB41a cells was evenly labeled. The high contrast and the excellent preservation of the ultrastructure were striking as even fine filopodia could be traced reliably without the loss of the intracellular fine structure.

**Fig 7:**
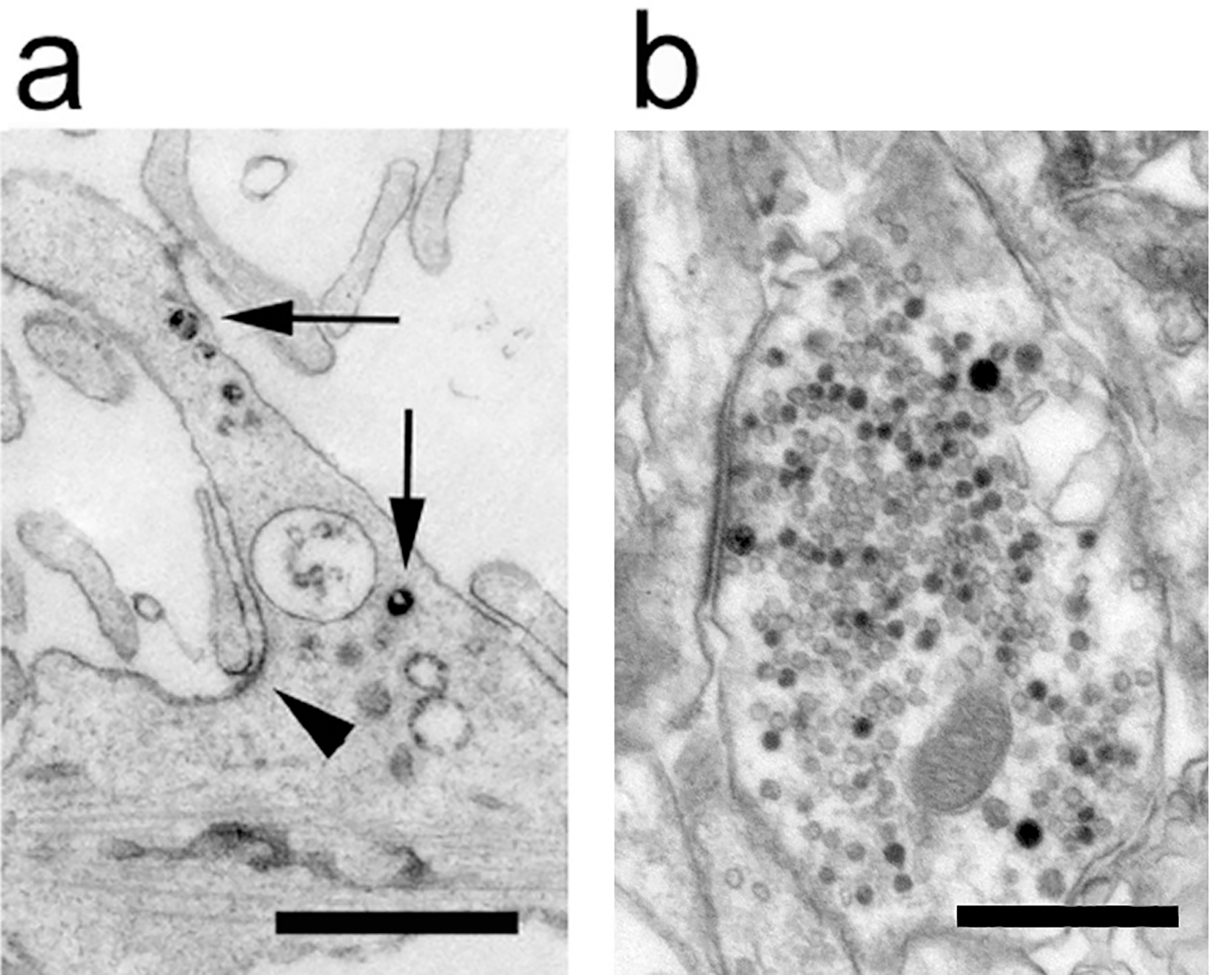
Neuronal cells expressing a fusion protein of eHRP and synaptotagmin 1 (eHRPsyt1). (a) Numerous vesicles (arrows) of various sizes are HRP-positive in a young cultured hippocampal neuron. At some locations eHRPsyt1 can be found at the plasma membrane (arrowhead). Bar = 200 nm. (b) An electron micrograph of an eHRPsyt1-positive presynaptic bouton. Note the numerous synaptic vesicles distributed throughout the synaptic vesicle cluster. Some labeled synaptic vesicles are also docked at the active zone (to the left). Bar = 200 nm.

Encouraged by the success of this approach, we targeted eHRP to synaptic vesicles (SVs) as a third example for cell organelle-specific targeting. We build a fusion protein of eHRP and the SV protein, synaptotagmin 1 (syt1) such that eHRP resided inside the lumen of SVs. After transfection of eHRP_syt1 into NB41a cells numerous small labeled vesicles were evident in the EM (Fig 7A). When we infected cultured hippocampal neurons with eHRP_syt1 numerous SVs were labeled in synaptic boutons (Fig 7B).

### Quintuple labeling of a single sample

The described experiments showed that cell-organelle-specific targeting is an elegant technique. With our three eHRP fusion proteins we are able to achieve triple labeling with genetically encoded labels. This number can be further increased by targeting eHRP to additional cell organelles for example to mitochondria. However, when relying on cell organelles alone, the number of unique labels is limited to the number of cell organelles. For true multilabeling the number of unique labeling patterns, however, should exceed the number of cell organelles.

**Fig 8:**
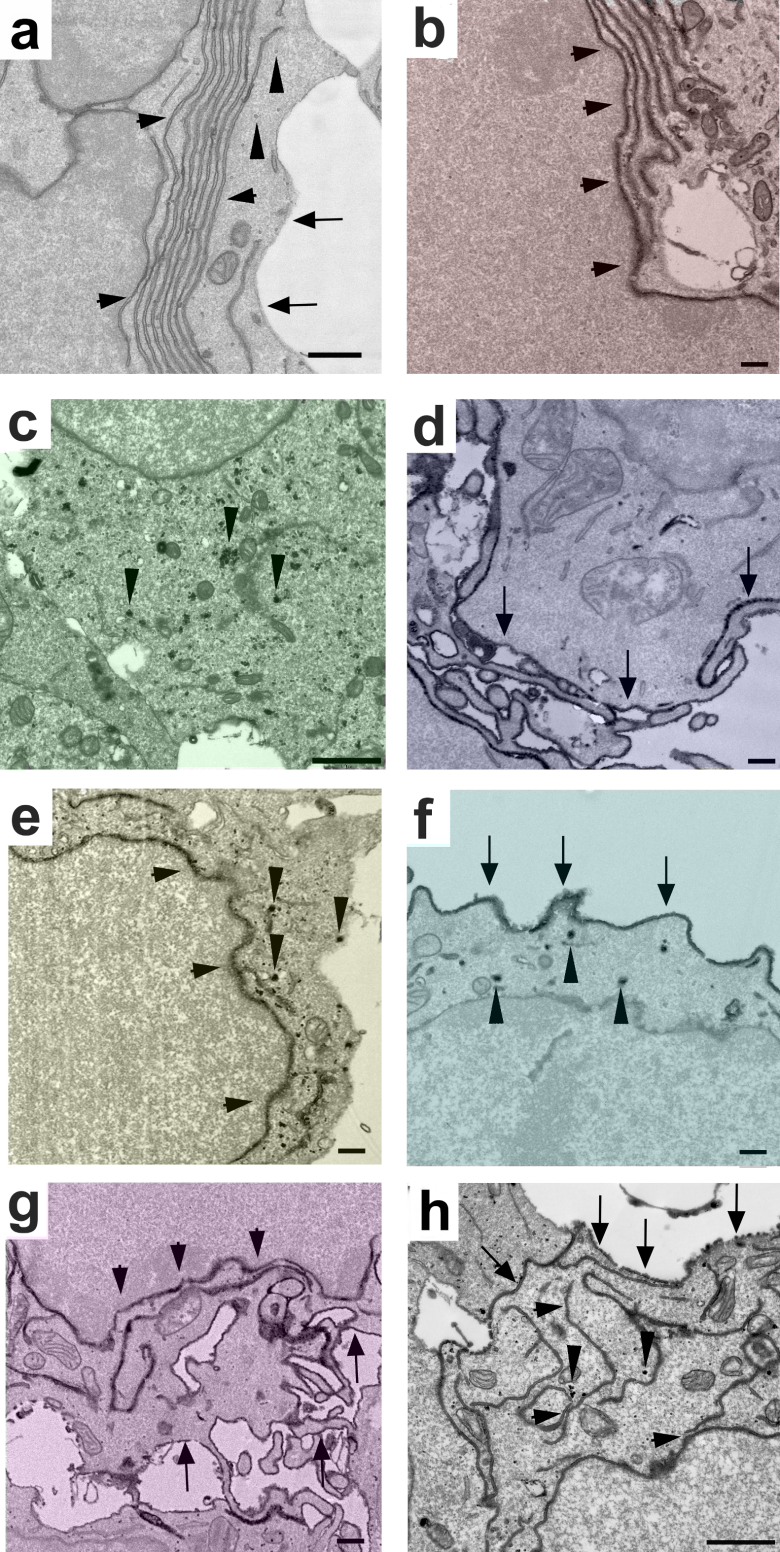
Quintuple labeling of neuroblastoma cells in a single sample. Each unique label and/or combination of labeling corresponds to a unique color. The three single labelings are in red, green and blue. The double labelings are in yellow, turquoise, and magenta. The triple labeling is in white. (a) An unlabeled cell. The horizontal arrowheads point at the endoplasmic reticulum, the vertical arrowheads at small vesicles with an electron-lucent lumen. The arrows demarcate the plasma membrane. (b) A cell expressing ER-targeted eHRP (arrowheads). (c) Expression of eHRPsyt1. Note the numerous small eHRP-positive vesicles in the cytosol (e.g. arrowheads). (d) Single expression of eHRP targeted to the plasma membrane. The arrows point at the labeled surface of the cell. (e) Double expression ER-targeted eHRP and eHRPsyt1. The horizontal arrowheads point to the labeled ER and the vertical arrowheads to vesicle labeling. (f) Double labeling with eHRP targeted to the plasma membranes and eHRPsyt1. The arrows point at the labeled cell surface and the arrowheads to labeled vesicles. (g) Double labeling of ER-targeted eHRP and eHRP targeted to the plasma membrane. Again, the arrows indicate the labeled plasma membrane and the horizontal arrowheads the labeled ER. (h) Triple labeling with all three fusion proteins. Horizontal arrows mark the labeled ER, vertical arrows labeled vesicles, and arrows point at the labeled cell surface. Bars = 500 nm in (a), 200 nm in (b), 1 μm in (c), 200 nm in (d), 500 nm in (e), 200 nm in (f), 200 nm in (g), 1 μm in (h).

One way to increase the number of unique labels beyond the number of cell organelles may be the combinatorial expression for example via ER- and SV-targeted eHRP. Now two fusion proteins could create three distinct labels. Armed with our three genetically encoded labels for EM, we tested whether such a strategy is feasible. We transfected HEK cells with a cocktail of individual created DNA complexes of the three constructs and left it to chance which plasmid or plasmids entered individual cells. This strategy theoretically results in seven different combination of labeling: three labels where only one cell organelle type is labeled, three labeling patterns in which two types of cell organelles are labeled and one in which all three cell organelles are labeled. Fig 8 presents all seven labeling patterns imaged in a single sample. All labeling patterns were clearly distinguishable from each other: single labeling with ER-targeted, SV-targeted, or plasma membrane-targeted eHRP (Fig 8B–8D), double labeling of ER- and SV targeted eHRP (Fig 8E), SV- and plasma membrane-targeted eHRP (Fig 8F), ER- and plasma membrane-targeted eHRP (Fig 8G), and finally a triple labeling with all three fusion proteins (Fig 8H). These experiments show that combinatorial expression of various cell-organelle targeted eHRP fusion proteins is a feasible technique for creating large numbers of uniquely identifiable genetically encoded labels in the EM.

With the introduction of combinatorial cell organelle-specific multilabeling, we filled a significant gap in our EM tool sets. The technique will likely further advance the development of modern genetic strategies in EM on which fluorescence microscopy has been thriving for decades. For the development of combinatorial cell organelle-specific multilabeling, we first identified a set of cell organelles that label cells without any overlap in labeled subcellular structures. Indeed, the three chosen organelles showed no overlap as PM-targeted eHRP labeled the outside of the cell membrane, ER-targeted eHRP was confined inside the ER, and eHRPsyt1 was localized to small vesicles. This complete separation of labeling patterns was the basis for the generation of unique labeling patterns when using combinatorial expression.

With combinatorial expression, the number of unique labeling patterns increases exponentially with the number of labeled organelles. In contrast, when labeling only individual organelles the number of labels increases linearly. In the linear approach, the number of organelles that can be labeled unambiguously without overlap is rather limited and therefore is insufficient for genetic strategies that require true multilabeling. We, however, demonstrated seven distinct labeling patterns with just three eHRP constructs. Labeling one additional cell organelle (for example mitochondria) further increases distinguishable labeling patterns and creates up to 15 labels. With such high number of labels many new genetic strategies for EM can be designed and analyzed in a single specimen. For example, the overexpression, knock-down, the rescue of the knock-down, rescue with individual mutants can now be imaged and analyzed on a single thin section in the EM.

True multilabeling is of particular interest when tracing various synaptic connections in connectome studies. Recent reports already demonstrated that targeting a label to specific cell organelles is an elegant strategy for the ultrastructural analysis of local neuronal circuits. Joesch and co-workers placed APEX2 and a HRP variant under the control of two distinct cell type specific promoters [15]. In Drosophila, HRP targeted to the cell membrane and APX targeted to mitochondira was used to study the connectivity of the chromatic pathway in the visual system [16]. Cell organelle specific targeting was also applied by using the miniSOG’s in Drosophila [17]. These studies and others were recently reviewed by Shigemoto and Joesch [18]. With combinatorial expression of cell organelle type-specific eHRP seven or more different neuron types can be labeled which undoubtley will further advance our understanding of neuronal connectivity.

For such an approach, cell organelles should be chosen carefully to ensure simultaneous presence of all organelles in neuronal processes. This is the case for the cell membrane, the ER, and mitochondria. SV’s, however prevalent in axons, are sparse in dendrites and are not useful for the identification of dendritic spines. Also, because neuronal processes are small it is possible that not all labeled cell organelles are present in a single section. This limitation can be overcome by using 3D reconstruction which has become a standard technique in connectivity studies.

Cell organelle specific labeling with HRP has been applied in the past. A classic study used HRP to monitor trafficking in and out of the Golgi apparatus at the EM level [19]. Later, a fusion protein of P-selectin and HRP was used to study secretory vesicles [20]. In Drosophila, HRP was fused to wingless to monitor the diffusion and spread of a secretory signal [21]. HRP has also been applied in neurons [14] and later the distribution of synapses on dendrites during development [22], and the labeling of vesicles in neurons has been reported [23–25]. Despite the success of these studies, HRP did not take of as a genetically encoded label for EM. One likely reason has been the unreliable detection of wild-type HRP when expressed in mammalian cells. Previous studies applied high sensitive developing techniques to remedy this shortcoming but those techniques also have the tendency to introduce background and compromise the fine structure. eHRP’s high enzyme activity overcomes these limitations. This increase in sensitivity also allows for the reliable labeling of various cell organelles with a high signal to noise ratio. As such, the directed evolution of eHRP was the very basis for the successful development of combinatorial cell organelle-specific multilabeling.

When comparing eHRP and eGFP detection it appeared that eHRP was more sensitive because many cells showed a dark HRP reaction product while eGFP labeling of the same cell was rather weak. The same is likely for APEX2, since the sensitivity of APEX2 is close to WT HRP [8], but eHRP originates from HRP mutants that exceed the sensitivity of WT HRP more than a 100 fold. Also, APEX2 lacks contrast in the EM which motivated the introduction of an additional reduction step before osmium fixation for a reliable visualization [15]. Such steps are unnecessary with eHRP. Taken together, these properties make eHRP the most sensitive genetically encoded label for EM currently available.

Just like HRP, eHRP is not detectable when expressed in the cytosol. This is not of any concern for the presented multilabeling approach because eHRP reaction product would occlude the fine structure of the cytosol and the other labels. But for proximity labeling in the cytosol for proteomic mapping [26] eHRP is not suitable. Split-HRP, however, is an alternative approach to study protein–protein interactions [27]. For this technique the combination of mutations of eHRP may be useful to increase the sensitivity of split HRP.

Early in our project, we noted that the various mutations may not simply additive. For example, the N175S mutation in the 13A7-N175S variant inhibited HRP’s activity in mammalian cells while in the 10G5 variant it did not. Similarly, the single N175S mutations in WT HRP increased HRP’s activity when expressed in brain [15]. It appears that the right combination of mutations is more important for enhancing HRP’s catalytic activity then individual mutations.

In terms of technical aspects, it should be noted that eHRP is compatible with standard HRP histochemistry. In other words, tissues and cells can be fixed with glutaraldehyde, and only DAB and very low concentrations of hydrogen peroxide (as low as 0.0003%) are needed for the visualization in the LM and EM. This standard and conventional technique ensures the preservation of the fine structure at the highest level and is compatible with labeling of large tissue blocks the same way wild-type HRP has been used in tract tracing studies for decades.

Beyond EM, eHRP is suitable for high resolution light and fluorescence microscopy. In this report, we demonstrated the use of eHRP in combination with eGFP in correlative live imaging light-electron microscopy, a technique essential to link physiology with molecular biology and morphology. But even without the co-expression of eGFP, eHRP can be visualized with the fluorescent AmplexRed for high resolution imaging. For example, high resolution imaging of the distribution of cell organelles followed by EM analysis are now easily achievable.

HRP has always been a very versatile label for a variety of techniques in structural biology and beyond. With the new capability of using genetically encoded eHRP it is likely that additional applications will emerge. As an example, we recently exploited eHRP for the local generation of oxygen radicals. eHRPsyt1 was targeted to the lumen of SVs and when a SV fused with the plasma membrane, extracellular hydrogen peroxide was cleaved and the radicals rendered the released SV dysfunctional. With this technique it was possible to dissect SV cycling in physiological experiments [28].
